# Quantification of myocardial strain assessed by cardiovascular magnetic resonance feature tracking in healthy subjects—influence of segmentation and analysis software

**DOI:** 10.1007/s00330-020-07539-5

**Published:** 2020-12-04

**Authors:** Carolin Lim, Edyta Blaszczyk, Leili Riazy, Stephanie Wiesemann, Johannes Schüler, Florian von Knobelsdorff-Brenkenhoff, Jeanette Schulz-Menger

**Affiliations:** 1 Working Group on Cardiovascular Magnetic Resonance, Experimental and Clinical Research Center, a Joint Cooperation Between the Charité – Universitätsmedizin Berlin, Department of Internal Medicine and Cardiology and the Max-Delbrueck Center for Molecular Medicine, and HELIOS Klinikum Berlin Buch, Department of Cardiology and Nephrology, Berlin, Germany; 2 DZHK (German Center for Cardiovascular Research), Partner Site, Berlin, Germany; 3 Berlin Ultrahigh Field Facility at the Max-Delbrueck Center for Molecular Medicine, Berlin, Germany; 4 Department of Cardiology, Clinic Agatharied, Ludwig-Maximilians - University München, Hausham, Germany

**Keywords:** Magnetic resonance imaging, Left ventricular function, Software, Myocard, Healthy volunteers

## Abstract

**Objectives:**

Quantification of myocardial deformation by feature tracking is of growing interest in cardiovascular magnetic resonance. It allows the assessment of regional myocardial function based on cine images. However, image acquisition, post-processing, and interpretation are not standardized. We aimed to assess the influence of segmentation procedure such as slice selection and different types of analysis software on values and quantification of myocardial strain in healthy adults.

**Methods:**

Healthy volunteers were retrospectively analyzed. Post-processing was performed using CVI^42^ and TomTec. Longitudinal and radial_Long axis (LAX)_ strain were quantified using 4-chamber-view, 3-chamber-view, and 2-chamber-view. Circumferential and radial_Short axis (SAX)_ strain were assessed in basal, midventricular, and apical short-axis views and using full coverage. Global and segmental strain values were compared to each other regarding their post-processing approach and analysis software package.

**Results:**

We screened healthy volunteers studied at 1.5 or 3.0 T and included 67 (age 44.3 ± 16.3 years, 31 females). Circumferential and radial_SAX_ strain values were different between a full coverage approach vs. three short slices (− 17.6 ± 1.8% vs. − 19.2 ± 2.3% and 29.1 ± 4.8% vs. 34.6 ± 7.1%). Different analysis software calculated significantly different strain values. Within the same vendor, different field strengths (− 17.0 ± 2.1% at 1.5 T vs. − 17.0 ± 1.7% at 3 T, *p* = 0.845) did not influence the calculated global longitudinal strain (GLS), and were similar in gender (− 17.4 ± 2.0% in females vs. − 16.6 ± 1.8% in males, *p* = 0.098). Circumferential and radial strain were different in females and males (circumferential strain − 18.2 ± 1.7% vs. − 17.1 ± 1.8%, *p* = 0.029 and radial strain 30.7 ± 4.7% vs. 27.8 ± 4.6%, *p* = 0.047).

**Conclusions:**

Myocardial deformation assessed by feature tracking depends on segmentation procedure and type of analysis software. Circumferential_SAX_ and radial_SAX_ depend on the number of slices used for feature tracking analysis. As known from other imaging modalities, GLS seems to be the most stable parameter. During follow-up studies, standardized conditions should be warranted.

**Trial registration** Retrospectively registered

**Key Points:**

*• Myocardial deformation assessed by feature tracking depends on the segmentation procedure.*

*• Global myocardial strain values differ significantly among vendors.*

*• Standardization in post-processing using CMR feature tracking is essential.*

**Supplementary Information:**

The online version contains supplementary material available at 10.1007/s00330-020-07539-5.

## Background

Quantification of myocardial deformation applying myocardial strain is of growing interest in cardiovascular magnetic resonance (CMR). For a few years, it has been applied in research, and different vendors have developed post-processing tools [1].

Myocardial strain allows quantitative measurement of global but also regional myocardial function and deformation offering additional information beyond ejection fraction [2, 3]. It enables early detection of subclinical myocardial dysfunction in patients with ischemic and non-ischemic heart disease and in preserved ejection fraction without wall motion abnormalities [2, 4–14].

Left ventricular deformation can be quantified in three dimensions: longitudinal and circumferential strain which show ventricular shortening in longitudinal and circumferential directions (negative strain) and radial strain that characterizes wall thickening (positive strain) [15].

Assessment of myocardial regional function is well known in echocardiography using speckle tracking [12, 15, 16] but is also increasingly investigated in CMR using different techniques, such as strain encoding (SENC) [17, 18], displacement encoding (DENSE) [19], and tagging [17, 18, 20–22]. Feature tracking is a tool which in contrast to the methods mentioned above enables post-processing analysis of myocardial strain based on routine steady-state free precession (SSFP) cine images as acquired for the assessment of left ventricular (LV) function and volume [8, 16, 23]. It avoids acquisition of additional images and saves time [23]. Pre-existing contours for calculation of LV function can be used for strain analysis making it a timesaving method. For those reasons, feature tracking seems to be a beneficial tool, e.g., for follow-up examinations.

Even though publications regarding CMR strain analysis exist, standards for image acquisition and interpretation are still not established. Different vendors and different analysis procedures such as slice selection procedures, even within the same software, can heavily influence deformation values. This may lead to uncertainties in comparison and interpretation of data. We aimed to analyze the influence of segmentation procedure such as slice selection on values of quantification of myocardial strain in healthy adults. Additionally, we intended to analyze the influence of different software packages and to provide regional strain quantification.

## Methods

### Study population

We retrospectively screened 243 truly healthy subjects, who were prospectively examined in former studies [24–28]. Exclusion criteria were known cardiovascular risk factors, any pre-existing diseases or medications, impaired LV ejection fraction (LVEF) (< 55%), or pathological findings in 12 lead ECG or CMR. Incomplete CMR data for feature tracking analysis led to exclusion. That included lack of long-axis (LAX) or short-axis (SAX) slices (*n* = 137) or variable number of cardiac phases (*n* = 41). The ethics committee approved all studies. Informed written consent was obtained in concordance with the Helsinki Declaration.

### CMR acquisition

CMR was performed at 1.5-T and 3-T scanners. At 1.5 T (Magnetom Avanto), a 12-channel radio frequency coil was used and at 3 T (Magnetom Verio, both Siemens Healthineers) a 32-channel radio frequency coil. SSFP cine images were acquired during repeated breath-holds for LV in 4-chamber-view (4CV), 3-chamber-view (3CV), 2-chamber-view (2CV), and at least three SAX slices (SAX full coverage and/or three SAX slices in basal, midventricular, and apical plane). Recently, detailed sequence parameters were published [24–29]: at 1.5 T: repetition time 2.8 ms, slice thickness 6 mm, flip angle 80 degrees, echo time 1.2 ms, field of view 276 × 340 mm^2^, matrix 156 × 192, voxel size 1.4 × 1.4 × 7 mm, 30 cardiac phases; and at 3 T: repetition time 3.1 ms, slice thickness 6 mm, flip angle 45 degrees, echo time 1.3 ms, field of view 276 × 340 mm^2^, matrix 156 × 192, voxel size 1.4 × 1.4 × 7 mm, 30 cardiac phases.

Two independent experienced readers (SCMR level III) performed the visual evaluation of the cine images.

LV function and volumes were quantified in a whole SAX stack according to the recommendation of the SCMR [30] applying CVI^42^ software (Version 4.1.2, Circle Cardiovascular Imaging Inc.). Endo- and epicardial contours were manually drawn in end-diastolic and end-systolic phase. Papillary muscles were excluded from the LV volume.

### Feature tracking

**Fig. 1:**
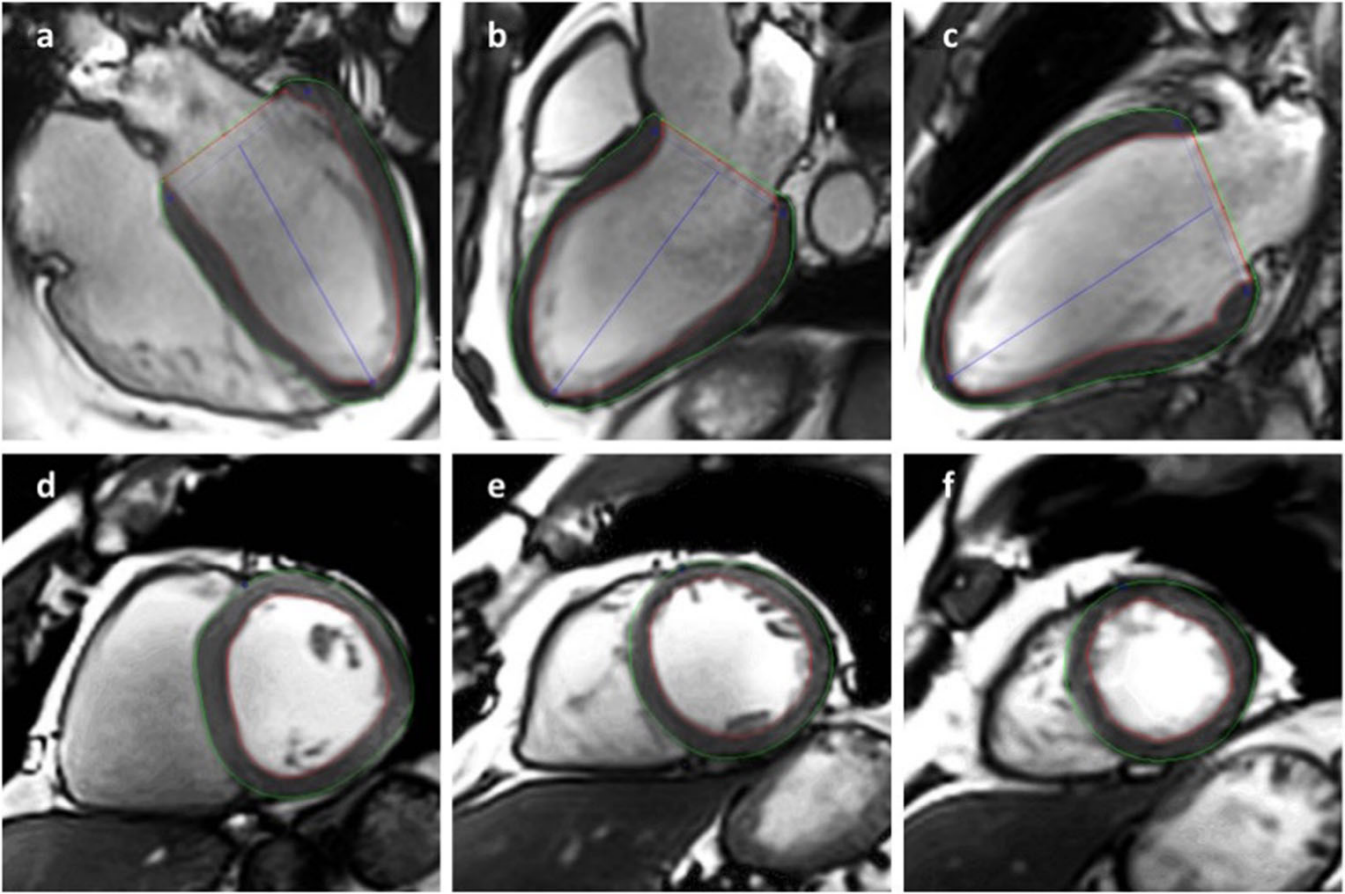
Post-processing using 2D strain analysis by CVI^42^. Endo- (red) and epicardial (green) contours were manually drawn in end-diastolic phase in long axis (**a**–**c**) and short axis (**d**–**f**). 4-chamber-view (**a**), 3-chamber-view (**b**), and 2-chamber-view (**c**) were included in long-axis strain analysis. For short-axis strain, contours were drawn in three short-axis slices: basal (**d**), midventricular (**e**), and apical (**f**)

**Fig. 2:**
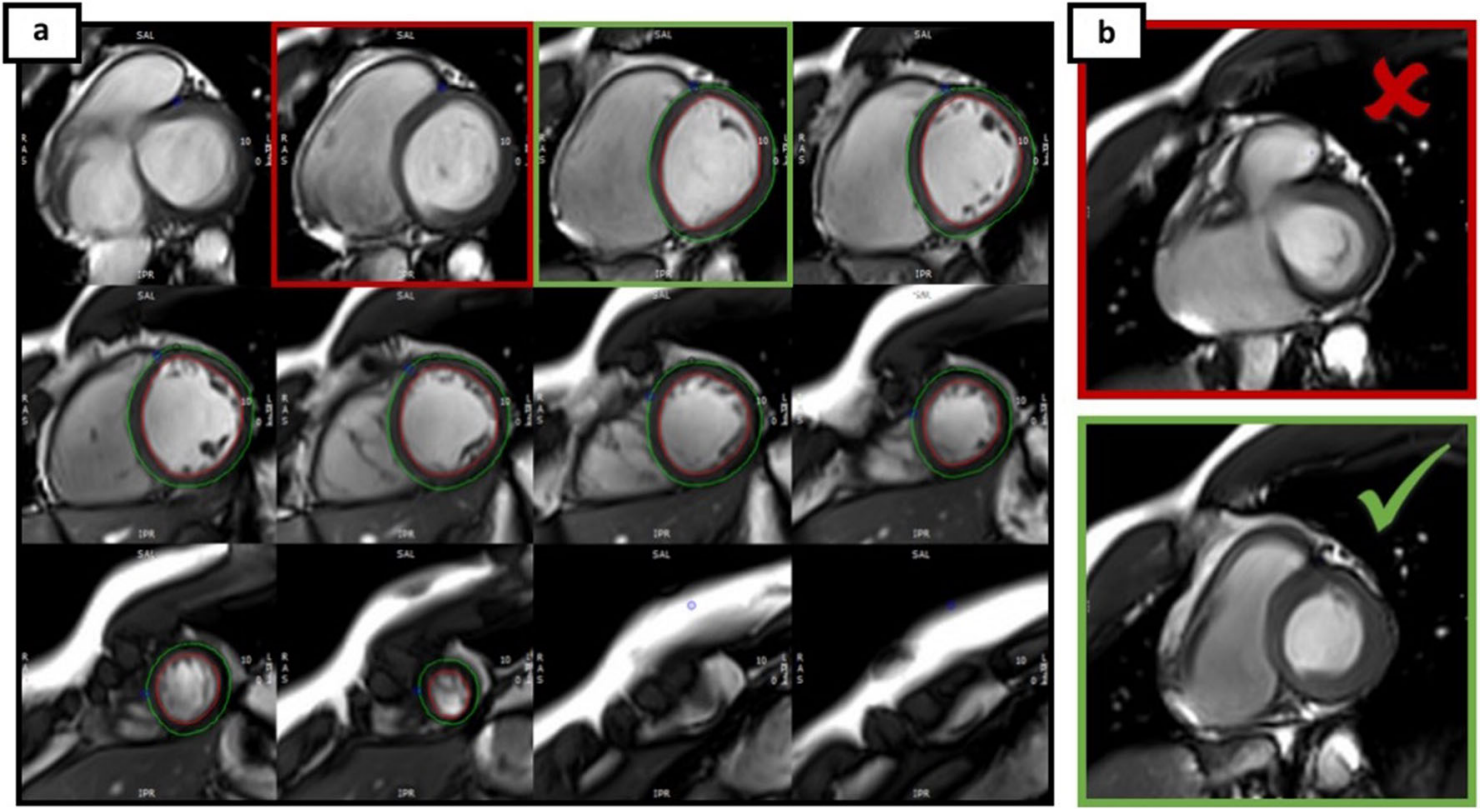
Strain analysis using full coverage (CVI^42^). Endo- and epicardial contours were drawn in end-diastolic phase (**a**). If LVOT was visible in end-systolic phase (**b**, marked red), slices were excluded. The first slice used for analysis was chosen as the most basal slice that did not show LVOT in any end-diastolic (**a**, marked green) and end-systolic phase (**b**, marked green)

**Fig. 3:**
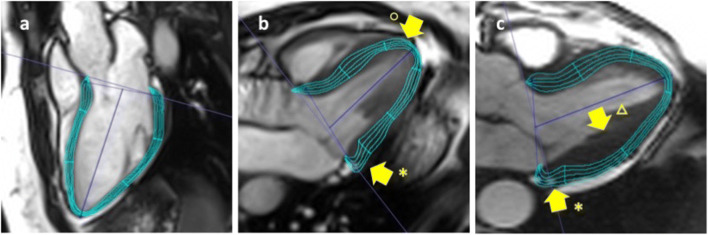
Quality assessment for accurate tracking and correct segmentation applying CVI^42^. **a** Optimal segmentation. **b** and **c** show incorrect segmentations in 3-chamber-view: the basal inferolateral segments are relatively short (*) and the apical septal segment extends to apical lateral (°). Additionally, contours do not follow endocardial borders accurately (∆)

Feature tracking analysis was performed retrospectively using CVI^42^ software (prototype version 5.3.0, Circle Cardiovascular Imaging Inc.). Longitudinal strain and radial_LAX_ strain (RS) were assessed in three LAX views: 4CV, 3CV, and 2CV (Fig. 1). Circumferential strain (CS) and RS_SAX_ were analyzed using three SAX slices (basal, midventricular, and apical) in all subjects (Fig. 1). If available, strain was additionally assessed using a SAX full coverage (Fig. 2). Endo- and epicardial contours were manually drawn in end-diastolic phase, defined as the phase with the largest LV volume. End-diastolic phase had to be identical in all SAX and LAX slices of one subject. Trabeculae, papillary muscles, pericardium, and epicardial fat were consequently excluded from contouring. Left ventricular outflow tract (LVOT) was completely excluded in all SAX slices if seen in diastolic and/or systolic phases (Fig. 2). 2D strain analysis was assessed globally and segmentally for longitudinal, RS_LAX_, CS, and RS_SAX_ strain. Segmentation included both possibilities of slice selection (three slices versus the whole stack) and the segmentation of the left ventricle according to the AHA 17-segment model [31]. We excluded the apex (segment 17) from feature tracking analysis; so far, the 16 segment model was used. Tracking quality and segmentation were evaluated using software tools like mesh, boundaries, or myocardial points. If contours did not follow the epi- or endocardial borders correctly, delineation was retraced and adjusted. In case of remaining tracking issues, all corresponding segments were excluded. Also, incorrect segmentation (see Fig. 3) led to exclusion. Excluded segments were not considered for global strain assessment.

Strain results were compared between field strengths (1.5 T and 3 T) and between different numbers of SAX slices (three SAX slices versus full coverage) in CS and RS_SAX_, as well as RS between LAX and SAX analysis.

Bulls-eye plots visualizing segmental strain values were created using the Python package Matplotlib.

Global strain analysis was repeated by the same observer (intra-observer) and by a different observer (inter-observer) in the same randomly selected subjects (*n* = 10).

### Software comparison

**Fig. 4:**
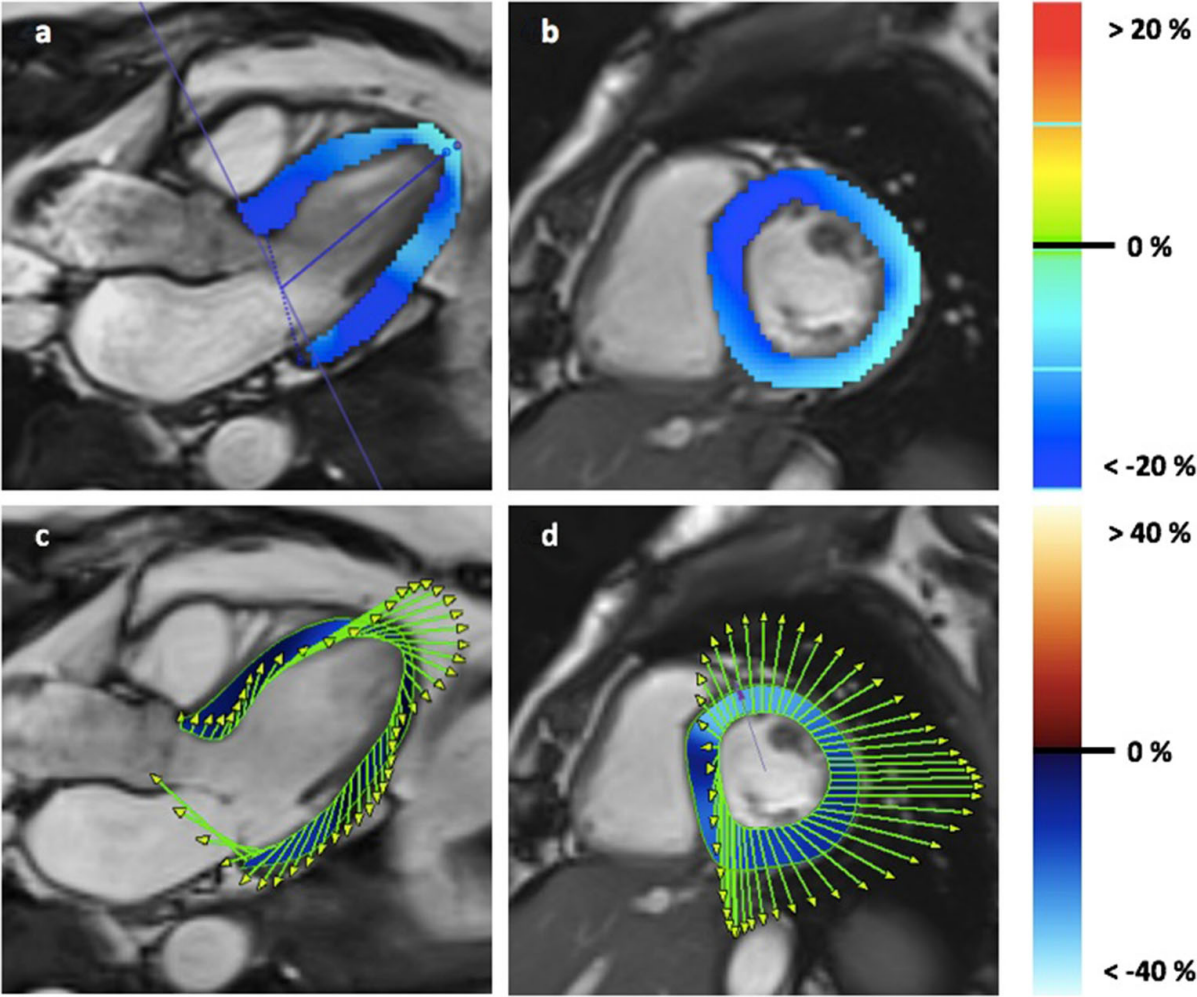
2D strain analysis of the left ventricle using different post-processing software. Strain was analyzed using CVI^42^ (**a**–**b**) and TomTec software (**c**–**d**). Longitudinal and radial_LAX_ strain were assessed in 4CV, 3CV (**a**, **c**), and 2CV; circumferential and radial_SAX_ strain were analyzed in basal (**b**, **d**), medial, and apical short-axis slice

All images were also analyzed with TomTec Image Arena (version 1.3.0.91, TomTec Imaging Systems GmbH) (Fig. 4). 4CV, 3CV, and 2CV were used for longitudinal and transversal (radial_LAX_) strain. CS and RS_SAX_ were assessed using three SAX slices (basal, midventricular, and apical). Endo- and epicardial contours were manually drawn in end-diastolic and end-systolic phases. Trabeculae and papillary muscles were excluded from analysis, as well as LVOT. Tracking quality was checked manually, specifically whether contours followed endo- and epicardial borders correctly and were adjusted if necessary. Myocardial strain was analyzed on a global and segmental level. Three LAX (4CV, 3CV, 2CV) and three SAX slices using the exact same slice number were considered for software comparison.

### Statistical analysis

Statistical analyses were performed using IBM SPSS Statistic version 23. We calculated mean values and standard deviation (SD) as well as median and interquartile ranges (IQR) for demographic parameters, LV function, and strain measurements. Volumes were indexed to body surface area (BSA) and height. The non-parametric Mann-Whitney *U* test for unpaired samples was used for comparisons of strain parameters between gender, analysis software, and field strength. Differences were considered to be statistically significant at *p* < 0.05. Intra- and inter-observer reproducibility were analyzed using intra-class correlation coefficient (ICC) and 95% confidence interval (CI). ICC was classified as poor (ICC < 0.4), good (ICC = 0.4–0.75), or excellent (ICC > 0.75) [1].

## Results

### Basic data

Sixty-seven healthy subjects (*n* = 36 at 1.5 T and *n* = 31 at 3 T) were included and analyzed (mean age 44.3 ± 16.3 years, *n* = 31 females). The proportion of men and age between the field strength groups was equalized. The 1.5 T group had 19 (52.8%), while the 3 T group accounted for 17 (54.8%) male subjects. Mean age was 45.0 ± 16.39 years at 1.5 T versus 43.48 ± 16.33 years at 3 T (*p* = 0.739).

All volunteers had normal LV function (LVEF 64.1 ± 4.2%) without wall motion abnormalities. Demographic parameters as well as LV function and volumes are summarized in Table 1. Seven subjects had to be excluded from 3D LV function analysis due to incomplete SAX package (*n* = 6) or artifacts (*n* = 1).

**Table 1:** Basic characteristics of the study population

	Mean ± SD	Median	Q1	Q3
Demographic parameters				
Gender (female | male)	31 | 36			
Age (years)	44.3 ± 16.3	45.0	28.0	59.0
Height (cm)	174.1 ± 8.6	173.0	168.0	180.0
Weight (kg)	74.4 ± 13.0	73.0	64.0	81.6
BMI (kg/m^2^)	24.6 ± 3.9	24.2	21.8	27.1
BSA (m^2^)	1.9 ± 0.2	1.9	1.8	2.0
HR (1/min)	72.8 ± 11.7	71.0	65.8	79.1
Systolic BP (mmHg)	128.1 ± 14.1	128.0	117.0	137.0
Diastolic BP (mmHg)	73.6 ± 11.7	75.0	68.0	79.0
LV function and volumes				
LVEF (%)	64.1 ± 4.2	64.0	60.4	67.2
LVEDV (ml)	139.9 ± 33.2	135.0	112.2	157.9
LVEDVI (ml/m^2^)	74.5 ± 15.1	76.1	62.6	83.3
LVEDVI (ml/cm)	0.8 ± 0.2	0.8	0.7	0.9
LVESV (ml)	51.1 ± 15.0	47.5	40.8	60.4
LVM (g)	101.0 ± 22.3	97.9	88.7	110.9
LVMI (g/m^2^)	53.7 ± 9.0	54.2	48.3	57.7
LVMI (g/cm)	0.6 ± 0.1	0.6	0.5	0.6
SV (ml)	89.2 ± 20.2	88.0	75.6	101.1
SVI (ml/m^2^)	47.5 ± 9.4	46.6	39.2	54.8
SVI (ml/cm)	0.5 ± 0.1	0.5	0.4	0.6

Data are shown as mean values ± standard deviation (SD), median, and interquartile ranges (Q1 and Q3)

*BMI* body mass index, *BSA* body surface area (Mosteller), *HR* heart rate, *BP* blood pressure, *LVEF* left ventricular ejection fraction, *LVEDV* left ventricular end-diastolic volume, *LVEDVI* left ventricular end-diastolic volume index, *LVESV* left ventricular end-systolic volume, *LVM* left ventricular mass, *LVMI* left ventricular mass index, *SV* stroke volume, *SVI* stroke volume index

### Feature tracking quality

In all 67 subjects, strain was analyzed in 4CV, 3CV, 2CV, and three SAX slices. Sixty-one subjects were additionally analyzed by CVI^42^ using a full coverage. Using CVI^42^, we could include 1020 segments (95.1%) for longitudinal strain and 1033 segments (96.4%) for RS_LAX_. In total, 1064 segments (99.3%) for RS_SAX_ and 1064 segments (99.3%) for CS were analyzed. In the SAX, strain analysis using SAX full coverage 966 segments (99.0%) from each of RS_SAX_ and CS could be included.

For analysis with TomTec, 1059 segments (98.8%) could be included for longitudinal strain, 1056 segments (98.5%) for RS_LAX_, 1071 segments (99.9%) for RS_SAX_, and 1070 segments (99.8%) for CS.

Reasons for exclusion were inaccurate tracking or incorrect segmentation.

### Influence of slice selection on circumferential and radial_SAX_ strain using CVI^42^

CS and RS_SAX_ measurements assessed by a stack of short axes covering the whole LV differ significantly from those assessed using three short axes: global CS − 19.2 ± 2.3% (median − 19.0%, IQR − 20.6 to − 17.9%) in 3 SAX vs. − 17.6 ± 1.8% (median − 17.7%, IQR − 18.6 to − 16.7%) in full coverage (*p* < 0.001) and global RS_SAX_ 34.6 ± 7.1% (median 33.4%, IQR 29.9–38.8%) in 3 SAX vs. 29.1 ± 4.8% (median 29.1%, IQR 26.2–31.9%) in full coverage (*p* < 0.001) (for details, see Table 2).

**Table 2:** Global strain values based on field strength, gender, and different post-processing software

		**Longitudinal strain (%)**						**Radial**_**LAX**_ **strain (%)**					
		**Mean ± SD**	**Median**		**Q1**	**Q3**	***p*** **value**	**Mean ± SD**		**Median**	**Q1**	**Q3**	***p*** **value**
CVI^42^		− 17.0 ± 1.9	− 17.0		− 18.4	− 15.6		29.1 ± 5.3		29.1	25.1	32.8	
	1.5 T	− 17.0 ± 2.1	− 17.0		− 18.4	− 15.3	0.845	29.1 ± 5.8		28.8	25.1	33.6	0.792
	3 T	− 17.0 ± 1.7	− 17.1		− 18.0	− 15.8		29.1 ± 4.6		29.4	26.3	32.5	
	Females	− 17.4 ± 2.0	− 17.7		− 18.4	− 15.8	0.098	30.7 ± 5.7		29.9	26.3	34.0	*0.033*
	Males	− 16.6 ± 1.8	− 16.6		− 18.0	− 15.3		27.8 ± 4.5		27.4	24.4	30.4	
TomTec		− 20.5 ± 2.7	− 20.2		− 22.6	− 18.8		70.1 ± 21.0		65.2	56.0	79.7	
	1.5 T	− 20.2 ± 2.3	− 19.8		− 22.1	− 18.3	0.114	77.8 ± 22.9		78.1	59.1	93.5	*0.002*
	3 T	− 20.8 ± 3.2	− 20.7		− 23.1	− 19.2		60.9 ± 14.1		60.1	52.6	72.2	
	Females	− 20.7 ± 3.2	− 20.3		− 23.2	− 19.3	0.268	74.9 ± 25.9		75.3	57.9	89.0	0.087
	Males	− 20.3 ± 2.2	− 19.9		− 22.4	− 18.7		66.1 ± 15.1		61.2	55.5	77.8	
		**Circumferential strain (%)**											
		**3 SAX**						**Full coverage**					**3 SAX vs. full coverage**
		**Mean ± SD**		**Median**	**Q1**	**Q3**	***p*** **value**	**Mean ± SD**	**Median**	**Q1**	**Q3**	***p*** **value**	***p*** **value**
CVI^42^		− 19.2 ± 2.3		− 19.0	− 20.6	− 17.9		− 17.6 ± 1.8	− 17.7	− 18.6	− 16.7		*< 0.001*
	1.5 T	− 19.6 ± 2.3		− 18.9	− 21.3	− 18.0	0.263	− 17.7 ± 1.8	− 17.7	− 18.8	− 16.6	0.855	*< 0.001*
	3 T	− 18.8 ± 2.2		− 19.0	− 20.5	− 17.6		− 17.6 ± 1.8	− 17.6	− 18.5	− 16.9		*0.012*
	Females	− 20.0 ± 2.2		− 20.0	− 21.5	− 18.4	*0.010*	− 18.2 ± 1.7	− 18.2	− 19.0	− 17.4	*0.029*	
	Males	− 18.6 ± 2.1		− 18.4	− 20.1	− 17.8		− 17.1 ± 1.8	− 17.4	− 18.5	− 16.2		
TomTec		− 20.7 ± 2.6		− 20.8	− 23.1	− 18.7							
	1.5 T	− 20.6 ± 2.4		− 20.5	− 22.5	− 18.6	0.389						
	3 T	− 20.9 ± 2.8		− 21.1	− 23.4	− 19.0							
	Females	− 21.0 ± 2.8		− 20.8	− 23.4	− 19.5	0.436						
	Males	− 20.5 ± 2.5		− 20.6	− 22.9	− 18.6							
		**Radial**_**SAX**_ **strain (%)**											
		**3 SAX**					**Full coverage**						**3 SAX vs. full coverage**
		**Mean ± SD**	**Median**	**Q1**	**Q3**	***p*** **value**	**Mean ± SD**	**Median**		**Q1**	**Q3**	***p*** **value**	***p*** **value**
CVI^42^		34.6 ± 7.1	33.4	29.9	38.8		29.1 ± 4.8	29.1		26.2	31.9		*< 0.001*
	1.5 T	36.0 ± 7.5	34.1	30.2	41.3	0.128	29.4 ± 5.1	29.6		25.9	32.3	0.665	*< 0.001*
	3 T	33.0 ± 6.2	32.6	28.9	37.3		28.7 ± 4.5	28.7		26.6	31.1		*0.006*
	Females	36.9 ± 7.2	37.1	30.7	42.3	*0.014*	30.7 ± 4.7	29.7		28.2	32.9	*0.047*	
	Males	32.7 ± 6.4	31.6	29.6	36.2		27.8 ± 4.6	28.0		25.0	31.2		
TomTec		63.7 ± 16.0	64.1	51.1	74.9								
	1.5 T	57.4 ± 12.7	56.0	47.1	69.6	*0.001*							
	3 T	71.0 ± 16.4	70.2	60.5	85.3								
	Females	67.8 ± 16.4	70.8	58.1	77.6	*0.022*							
	Males	60.2 ± 15.0	57.2	49.8	65.2								

Global strain values are given as mean ± standard deviation (SD), median, and interquartile range (Q1 and Q3). Significant differences are shown in italics

*LAX* long axis, *SAX* short axis

Using three SAX slices, no differences were found for global strain measurements between 1.5 T and 3 T: global CS − 19.6 ± 2.3% (median − 18.9%, IQR − 21.3 to − 18.0%) at 1.5 T vs. − 18.8 ± 2.2% (median − 19.0%, IQR − 20.5 to − 17.6%) at 3 T (*p* = 0.263) and for global RS_SAX_ 36.0 ± 7.5% (median 33.4%, IQR 29.9–38.8%) at 1.5 T vs. 33.0 ± 6.2% (median 32.6%, IQR 28.9–37.3%) at 3 T (*p* = 0.128). However, some segmental strain values differed significantly between field strengths for both CS and RS_SAX_ (for details, see supplemental material online additional file 1).

Using SAX full coverage, both global and segmental strain values did not show any significant difference between field strengths: CS − 17.7 ± 1.8% (median − 17.7%, IQR − 18.8 to − 16.6%) at 1.5 T vs − 17.6 ± 1.8% (median − 17.6%, IQR − 18.5 to − 16.9%) at 3 T (*p* = 0.85) and global RS_SAX_ 29.4 ± 5.1% (median 29.6%, IQR 25.9–32.3%) at 1.5 T vs 28.7 ± 4.5% (median 28.7%, IQR 26.6–31.1%) at 3 T (*p* = 0.665) (for details, see supplemental material online additional file 1).

In both, three selected slices and a whole SAX stack global circumferential and radial_SAX_ strain differed significantly between genders (for details, see Table 2). Gender-related strain values are visualized in the supplemental material additional file 2.

### Assessment of radial strain in long- and short-axis views

Global radial strain acquired in LAX (radial_LAX_) versus SAX (radial_SAX_) differed significantly: global radial_LAX_ 29.1 ± 5.3% (median 29.1%, IQR 25.1–32.8%) versus global radial_SAX_ 34.6 ± 7.1% (median 33.4%, IQR 29.9–38.8%) (*p* < 0.001).

### Longitudinal strain using CVI^42^

**Fig. 5:**
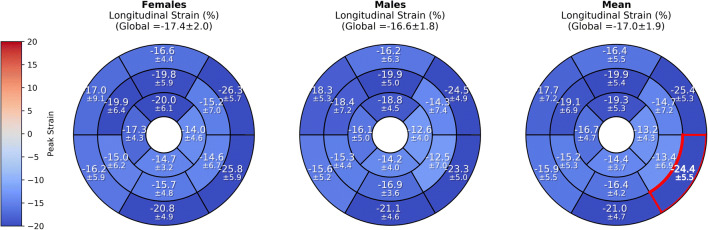
Gender-related mean values for longitudinal strain using CVI^42^. Segmental values are provided as mean (in %) ± standard deviation in a bulls-eye plot according to the AHA segment model [31]. Segment 5 (marked red) differed between genders (*p* = 0.048)

Longitudinal strain did not show any significant difference for both global and segmental strain measurements between 1.5 T and 3 T: − 17.0 ± 2.1% (median − 17.0%, IQR − 18.4 to − 15.3%) vs. − 17.0 ± 1.7% (median − 17.1%, IQR − 18.0 to − 15.8%) (*p* = 0.845 accordingly). No significant differences have been found between females and males: − 17.4 ± 2.0% (median − 17.7%, IQR − 18.4 to − 15.8%) and − 16.6 ± 1.8% (median − 16.6%, IQR − 18.0 to − 15.3%) (*p* = 0.098). On a segmental level, only AHA segment 5 (basal inferolateral) showed a significant difference between genders: − 25.8 ± 5.9% (median − 26.7%, IQR – 30 to − 22.8%) in females versus − 23.3 ± 5.0% (median − 23.4%, IQR − 27.5 to − 18.4%) in males (*p* = 0.048). Segmental strain measurements for longitudinal strain are presented in Fig. 5.

### Software comparison

Strain measurements assessed with TomTec software were significantly different to those assessed with CVI^42^ (Table 3). GLS was − 17.0 ± 1.9% (median − 17.0%, IQR − 18.4 to − 15.6%) for CVI^42^ and − 20.5 ± 2.7% (median − 20.2%, IQR − 22.6 to − 18.8%) for TomTec (*p* < 0.001). Significant differences were also found for most segmental strain values (for details, see supplemental material online additional files 3–6).

**Table 3:** Global myocardial strain compared between different post-processing software

		CVI^42^				TomTec				*p* value
		Mean ± SD	Median	Q1	Q3	Mean ± SD	Median	Q1	Q3	
Global longitudinal strain (%)		− 17.0 ± 1.9	− 17.0 ± 1.9	− 17.0	− 18.4	− 20.5 ± 2.7	− 20.2	− 22.6	− 18.8	*< 0.001*
	1.5 T	− 17.0 ± 2.1	− 17.0 ± 2.1	− 17.0	− 18.4	− 20.2 ± 2.3	− 19.8	− 22.1	− 18.3	*< 0.001*
	3 T	− 17.0 ± 1.7	− 17.0 ± 1.7	− 17.1	− 18.0	− 20.8 ± 3.2	− 20.7	− 23.1	− 19.2	*< 0.001*
Global circumferential strain (%)		− 19.2 ± 2.3	− 19.0	− 20.6	− 17.9	− 20.7 ± 2.6	− 20.8	− 23.1	− 18.7	*0.001*
	1.5 T	− 19.6 ± 2.3	− 18.9	− 21.3	− 18.0	− 20.6 ± 2.4	− 20.5	− 22.5	− 18.6	0.076
	3 T	− 18.8 ± 2.2	− 19.0	− 20.5	− 17.6	− 20.9 ± 2.8	− 21.1	− 23.4	− 19.0	*0.001*
Global radial_SAX_ strain (%)		34.6 ± 7.1	33.4	29.9	38.8	63.7 ± 16.0	64.1	51.1	74.9	*< 0.001*
	1.5 T	36.0 ± 7.5	34.1	30.2	41.3	57.4 ± 12.7*	56.0	47.1	69.6	*< 0.001*
	3 T	33.0 ± 6.2	32.6	28.9	37.3	71.0 ± 16.4*	70.2	60.5	85.3	*< 0.001*
Global radial_LAX_ strain (%)		29.1 ± 5.3	29.1	25.1	32.8	70.1 ± 21.0	65.2	56.0	79.7	*< 0.001*
	1.5 T	29.1 ± 5.8	28.8	25.1	33.6	77.8 ± 22.9*	78.1	59.1	93.5	*< 0.001*
	3 T	29.1 ± 4.6	29.4	26.3	32.5	60.9 ± 14.1*	60.1	52.6	72.2	*< 0.001*

Global strain values are given as mean ± standard deviation (SD), median, and interquartile ranges (Q1 and Q3). Radial_SAX_ and circumferential strain were assessed using three short-axis slices (basal, midventricular, apical). Significant differences (*p* < 0.05) are shown in italics. * *p* < 0.05 between 1.5 T and 3 T within one software

Gender-related global strain values using TomTec are summarized in Table 2. Unlike differences in global RS_SAX_, GLS and global CS were not associated with gender.

### Intra- and inter-observer reproducibility (CVI^42^)

GLS reproducibility was as follows: ICC was 0.941 (95% CI 0.759–0.985) for intra-observer and 0.829 (95% CI 0.273–0.958) for inter-observer analysis. We observed an excellent intra- and inter-observer reproducibility across all global strain measurements (for details, see supplemental material 7). Intra-observer agreement was best for CS (ICC 0.977, 95% CI 0.907–0.994) and lowest for RS_LAX_ (ICC 0.930, 95% CI 0.715–0.983). Inter-observer agreement was best for radial_SAX_ strain (ICC 0.975, 95% CI 0.889–0.994) and lowest for longitudinal strain (ICC 0.829, 95% CI 0.273–0.958).

## Discussion

In this study, we aimed to increase knowledge about influencing factors on strain results obtained by CMR feature tracking. We focused on the segmentation procedure and on the comparison of software packages of two different vendors.

For the first time, we showed that CS and RS_SAX_ were dependent on the number of slices used for feature tracking analysis. Previous published studies considered a different number of slices for strain analysis making it difficult to compare strain values to each other. While some used one LAX and one midventricular SAX slice [20, 32, 33], others included two LAX and three SAX views [34, 35] or considered all three LAX views and a SAX full coverage [36]. The variation in analysis procedure like slice selection may lead to different quantitative results and consequently to uncertainties and difficulties in comparison and interpretation. Significant variations among vendors are already known in echocardiography and CMR-FT and this should be considered when performing serial studies [37]. A recent study by Liu et al compared 3D strain analysis (three LAX slices and SAX full coverage) with 2D analysis using one horizontal LAX and one midventricular SAX slice showing notable differences [38]. In our study, we detected differences for CS and RS_SAX_ between three SAX slices and full coverage using CVI^42^. Of note, both parameters were significantly higher using 3 SAX slices vs. full coverage; one should assume that partial volume effects, mainly effecting an apical slice, may influence the results. Furthermore, vendors may use a different way of pixel definition leading to a different boundary detection.

Radial strain assessed in LAX and SAX slices differed significantly. There is no broad experience in using radial_LAX_ strain yet, but when SAX slices are missing, assessment of radial strain in LAX can add information.

Among different types of post-processing software, both global and segmental strain values differed significantly. These findings indicate that strain values are not comparable between different software applications. Our findings in terms of differences among post-processing software packages are mostly in accordance with previous published data [1, 20, 38]. Barreiro-Pérez et al showed variability among different vendors (TomTec, CVI^42^, Medis, Medviso) in GLS and RS measurements, but not in CS [1]. In our study, strain values were significantly lower using CVI^42^, but these findings conform with previous studies [20, 38]. Cao et al compared different sequences and different post-processing software [20], detecting notable differences between all CMR techniques. However, the proper validation of most analysis procedures as well as absolute and objective reference values is yet to be established. While DENSE, SENC, and tagging, techniques for measuring three-dimensional motion and deformation, require dedicated sequences, feature tracking analysis is based on routine SSFP cine images. However, FT is based on contours only and does not follow intrinsic myocardial contraction. Moreover, the influence of field strengths seems to not be relevant. Schuster et al showed similar results for myocardial strain among 1.5 T and 3 T applying TomTec [32]. This agrees with our results since field strength did not influence global values of longitudinal, RS_LAX_, RS_SAX_, and CS strain using CVI^42^.

Reference values for CMR feature tracking analysis have been published, mainly focused on global left ventricular strain. Most studies performed feature tracking via TomTec [36, 39, 40]. Liu et al were the first to establish normal ranges for CVI^42^ using 3D strain analysis [38]. However, regional deformation was only acquired for CS. Regional assessment of myocardial strain is less validated, but may reveal further information compared to global values as single regions of the myocardium can be injured even though global strain is in normal range. We added knowledge on reference values for myocardial strain in healthy subjects using CVI^42^ and TomTec.

Unlike most studies showing greater deformation in females resulting in more negative strain [36, 39–42], we did not find gender-related differences for global longitudinal strain. The larger magnitudes of global CS in females having more negative strain values also agree with the findings reported by Andre et al and Peng et al [40, 41]. However, the higher global radial strain values in females contradict former findings [36, 40].

In accordance with our findings, CMR feature tracking has shown fair reproducibility in previous studies [34]. In fact, strain assessment is influenced by observer experience, but reproducibility may be optimized by training [43, 44]. Most studies indicate better reproducibility for global rather than segmental strain analysis with global CS being the most and global radial strain being the least reproducible measurement [20, 33, 35, 36, 42].

However, analysis methods throughout all studies were not standardized until now. CMR feature tracking–derived strain seems to be influenced by many factors including software package and the applied approach of image processing; thus, reference values should be derived from similar approaches. Currently, no gold standard exists. There is no defined “right” or “wrong” as in most of the publications that evaluate differences between post-processing software or sequences. But there is a need to understand that the application of different approaches may lead to different results.

CMR feature tracking is a promising tool that enables early detection of subtle myocardial dysfunction and prediction of major adverse cardiovascular events [5–7]. Standardization is needed if assessment of myocardial deformation including feature tracking should enter clinical routine.

### Limitations

This study is limited by a relatively small, but carefully and well-characterized healthy study cohort. As our analysis was performed retrospectively in prospectively enrolled volunteers, scan protocols were slightly different. This led to exclusion of 176 subjects due to incomplete CMR data. This may be preventable by a prospectively designed study, but our settings also reflect potential difficulties in clinical routine.

Our statistical analysis was only descriptive and exploratory. It indicates that differences among vendors or segmentation procedures may exist, but further validation remains necessary.

The CMR examinations performed at 1.5 T and 3 T did not contain the same subjects, but showed an equal distribution regarding gender and age. In accordance with our results, pre-existing studies have also shown that field strength does not influence global strain values [32].

CMR feature tracking is less validated for regional strain and radial_LAX_ strain, but they can presumably reveal different physiological mechanisms of the myocardium. Regional assessment is limited by inaccurate tracking or incorrect segmentation which may distort segmental strain values. We provide numbers, but long-term studies have to show the potential significance before CMR-FT may enter clinical routine.

## Conclusion

Myocardial deformation assessed by feature tracking depends on segmentation procedure and type of analysis software. Circumferential_SAX_ and radial_SAX_ depend on the number of slices used for feature tracking analysis. As known from other imaging modalities, GLS seems to be the most stable parameter. Standardized conditions should be considered.

## Supplementary information

ESM 1 (DOCX 1824 kb)

## Abbreviations

AHA: American Heart Association
CMR: Cardiovascular magnetic resonance
CS: Circumferential strain
GCS: Global circumferential strain
CV: Chamber view
GLS: Global longitudinal strain
GRE: Gradient echo sequence
LAX: Long axis
LGE: Late gadolinium enhancement
LV: Left ventricular
LVEDVI: Left ventricular end-diastolic index
LVEF: Left ventricular ejection fraction
LVOT: Left ventricular outflow tract
MOLLI: Modified look-locker inversion-recovery
RS: Radial strain
SAX: Short axis
SD: Standard deviation
SSFP: Steady-state free precession
