# Supplementary material for: Quantification of myocardial strain assessed by cardiovascular magnetic resonance feature tracking in healthy subjects—influence of segmentation and analysis software
Source: Eur Radiol. 2020 Dec 4;31(6):3962–72. doi: 10.1007/s00330-020-07539-5 (PMC8128822; doi:10.1007/s00330-020-07539-5)
Supplement: Supplementary file 1 — (DOCX 1824 kb) [file 330_2020_7539_MOESM1_ESM.docx]

**Supplemental material 1. Circumferential strain and radial_SAX_ strain in 1.5 T and 3 T using three short axis slices (3 SAX) and full coverage**

|  | Circumferential Strain [%] | | | | | | Radial_SAX_ Strain [%] | | | | | | |
| --- | --- | --- | --- | --- | --- | --- | --- | --- | --- | --- | --- | --- | --- |
|  | **3 SAX** | | | **Full coverage** | | | | **3 SAX** | | | **Full coverage** | | |
| **AHA-Segment** | **1.5 T** | **3 T** | **p value** | **1.5 T** | **3 T** | **p value** | | **1.5 T** | **3 T** | **p value** | **1.5 T** | **3 T** | **p value** |
| **1** | -24.4 ± 3.8 | -23.9 ± 4.0 | 0.678 | -22.8 ± 3.4 | -23.4 ± 3.0 | 0.412 | | 51.6 ± 17.3 | 49.4 ± 17.8 | 0.664 | 44.7 ± 12.6 | 46.8 ± 12.3 | 0.437 |
| **2** | -16.3 ± 3.3 | -16.4 ± 3.1 | 0.683 | -15.2 ± 3.4 | -15.4 ± 3.6 | 0.994 | | 25.7 ± 8.3 | 26.1 ± 8.2 | 0.706 | 23.3 ± 7.2 | 23.8 ± 8.3 | 0.965 |
| **3** | -11.8 ± 3.0 | -12.8 ± 4.6 | **0.033** | -11.3 ± 3.1 | -12.6 ± 3.6 | 0.105 | | 16.2 ± 5.4 | 18.8 ± 5.6 | **0.036** | 15.3 ± 5.6 | 18.0 ± 7.1 | 0.110 |
| **4** | -17.4 ± 4.5 | -17.3 ± 4.0 | 0.900 | -17.8 ± 3.1 | -17.8 ± 3.2 | 0.953 | | 29.6 ± 12.1 | 28.4 ± 10.9 | 0.772 | 29.3 ± 8.1 | 29.1 ± 8.4 | 0.912 |
| **5** | -22.2 ± 3.5 | -18.2 ± 3.4 | **< 0.001** | -19.3 ± 3.2 | -18.5 ± 2.3 | 0.182 | | 42.2 ± 12.4 | 30.1 ± 8.5 | **< 0.001** | 33.1 ± 8.8 | 30.5 ± 6.0 | 0.218 |
| **6** | -23.1 ± 4.3 | -21.2 ± 3.9 | 0.077 | -21.7 ± 3.3 | -20.8 ± 3.0 | 0.333 | | 46.4 ± 15.5 | 39.1 ± 11.7 | 0.097 | 40.7 ± 11.5 | 37.6 ± 9.5 | 0.340 |
| **7** | -19.8 ± 4.1 | -19.0 ± 4.1 | 0.597 | -18.8 ± 3.8 | -18.9 ± 3.1 | 0.415 | | 35.0 ± 12.9 | 32.7 ± 11.3 | 0.624 | 32.2 ± 12.0 | 31.8 ± 8.5 | 0.469 |
| **8** | -15.9 ± 3.9 | -17.3 ± 2.7 | 0.093 | -15.3 ± 3.3 | -15.5 ± 2.6 | 0.856 | | 24.7 ± 9.1 | 27.6 ± 6.7 | 0.083 | 23.3 ± 7.5 | 23.3 ± 5.8 | 0.928 |
| **9** | -14.4 ± 2.4 | -15.2 ± 2.7 | 0.211 | -12.8 ± 3.5 | -13.6 ± 3.5 | 0.551 | | 20.7 ± 5.1 | 22.6 ± 5.7 | 0.161 | 18.1 ± 6.7 | 19.8 ± 7.4 | 0.571 |
| **10** | -16.5 ± 3.6 | -17.1 ± 3.0 | 0.619 | -16.3 ± 3.3 | -16.4 ± 3.3 | 0.662 | | 26.1 ± 8.9 | 27.2 ± 7.6 | 0.576 | 25.5 ± 8.3 | 25.8 ± 7.7 | 0.640 |
| **11** | -20.6 ± 3.7 | -18.5 ± 3.6 | **0.022** | -19.7 ± 3.7 | -18.2 ± 3.4 | 0.179 | | 37.2 ± 10.9 | 30.8 ± 9.1 | **0.019** | 34.5 ± 10.7 | 30.2 ± 8.6 | 0.147 |
| **12** | -21.4 ± 3.9 | -18.8 ± 3.8 | **0.008** | -20.0 ± 3.5 | -19.4 ± 3.7 | 0.488 | | 39.9 ± 12.6 | 31.9 ± 9.8 | **0.009** | 35.5 ± 10.4 | 33.6 ± 10.6 | 0.478 |
| **13** | -22.2 ± 3.8 | -21.4 ± 3.9 | 0.337 | -20.8 ± 3.7 | -19.2 ± 3.5 | 0.069 | | 43.1 ± 13.6 | 40.4 ± 13.6 | 0.309 | 39.3 ± 11.9 | 33.9 ± 10.4 | 0.066 |
| **14** | -19.4 ± 4.3 | -19.4 ± 3.8 | 0.933 | -17.8 ± 4.0 | -18.1 ± 2.9 | 0.898 | | 34.3 ± 12.2 | 33.9 ± 10.7 | 0.913 | 30.4 ± 10.2 | 30.3 ± 7.9 | 0.988 |
| **15** | -23.1 ± 4.0 | -22.3 ± 4.2 | 0.531 | -21.5 ± 3.9 | -22.3 ± 3.7 | 0.497 | | 47.5 ± 15.8 | 43.6 ± 14.3 | 0.391 | 41.6 ± 12.5 | 44.0 ± 12.4 | 0.446 |
| **16** | -25.4 ± 3.6 | -23.5 ± 4.8 | 0.104 | -23.1 ± 4.1 | -21.9 ± 3.4 | 0.197 | | 56.6 ± 16.6 | 48.8 ± 16.1 | 0.097 | 48.1 ± 14.7 | 42.7 ± 11.5 | 0.189 |
| **Global** | -19.6 ± 2.3 | -18.8 ± 2.2 | 0.263 | -17.7 ± 1.8 | -17.6 ± 1.8 | 0.855 | | 36.0 ± 7.5 | 33.0 ± 6.2 | 0.128 | 29.4 ± 5.1 | 28.7 ± 4.5 | 0.665 |

Data are shown as mean values ± standard deviation (SD) according to the AHA-segment model [18]. Significant differences (p < 0.05) are highlighted in bold.

**Supplemental material 2.**

Gender-related strain values using CVI^42^

**
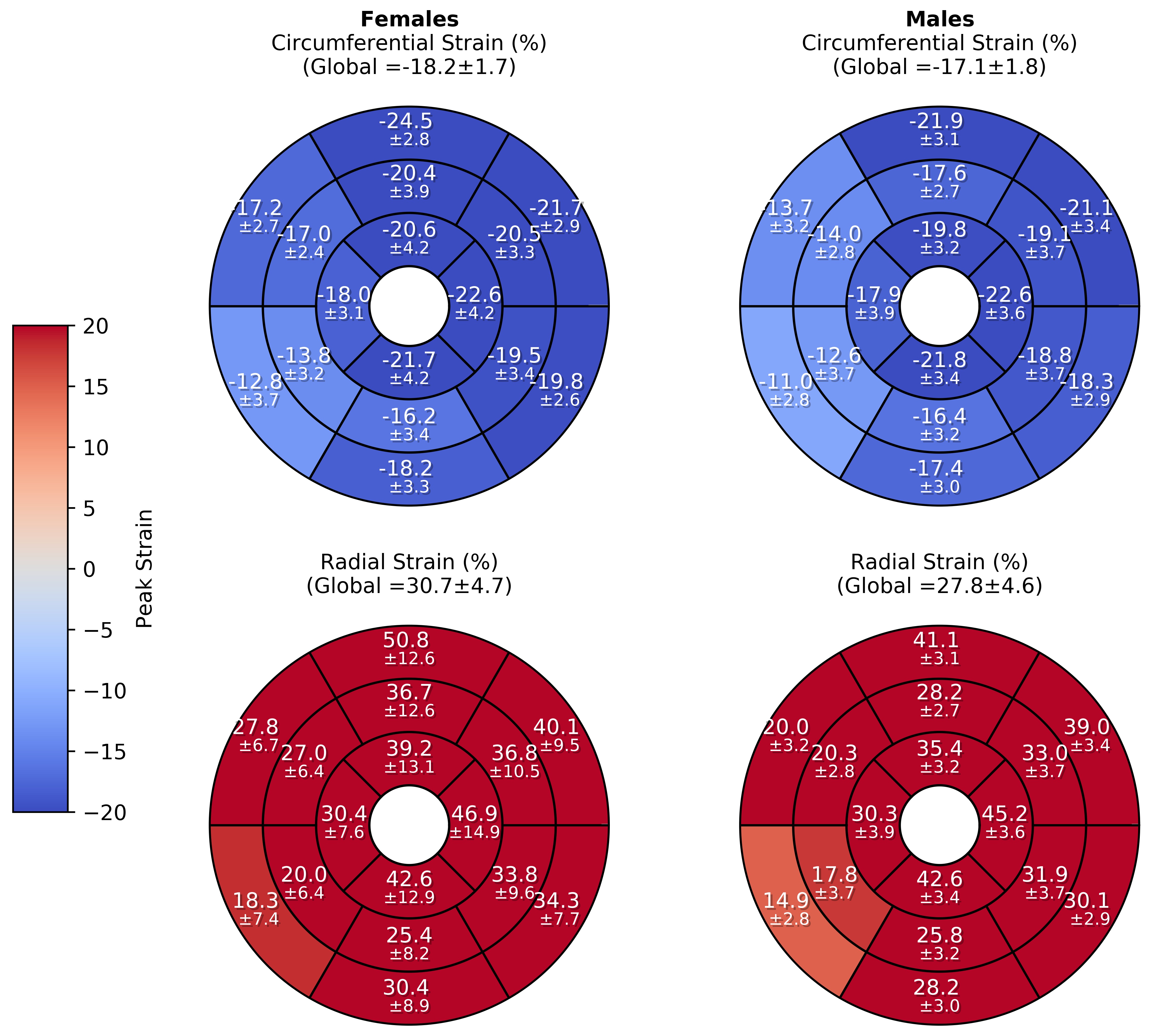
**

**Supplemental material 3.**

**Supplemental material 3. Software Comparison – Longitudinal Strain**

|  | CVI^42^ | | | TomTec | | |  |  |
| --- | --- | --- | --- | --- | --- | --- | --- | --- |
| **AHA-Segment** | **1.5 T** | **3 T** | **p value** | **1.5 T** | **3 T** | **p value** | **p value (1.5 T)** | **p value (3 T)** |
| **1** | -17.4 ± 4.3 | -15.1 ± 6.5 | 0.129 | -21.2 ± 10.4 | -16.7 ± 8.5 | **0.026** | **0.030** | 0.723 |
| **2** | -17.2 ± 8.2 | -18.4 ± 5.5 | 0.692 | -21.8 ± 6.2 | -18.6 ± 8.4 | 0.110 | **0.006** | 0.879 |
| **3** | -16.3 ± 5.8 | -15.4 ± 5.1 | 0.476 | -22.0 ± 7.4 | -20.2 ± 8.0 | 0.300 | **0.001** | **0.007** |
| **4** | -20.0 ± 5.0 | -22.2 ± 4.1 | 0.087 | -21.4 ± 7.9 | -19.4 ± 9.6 | 0.369 | 0.371 | 0.243 |
| **5** | -23.3 ± 6.0 | -25.8 ± 4.7 | 0.080 | -26.8 ± 9.2 | -30.6 ± 9.2 | 0.114 | 0.075 | **0.016** |
| **6** | -24.8 ± 5.2 | -26.0 ± 5.5 | 0.345 | -29.2 ± 9.9 | -30.9 ± 8.6 | 0.410 | **0.034** | **0.009** |
| **7** | -19.8 ± 4.9 | -20.0 ± 6.0 | 0.685 | -29.1 ± 11.1 | -32.0 ± 9.3 | 0.559 | **< 0.001** | **< 0.001** |
| **8** | -18.0 ± 7.6 | -20.4 ± 5.7 | 0.159 | -17.6 ± 9.0 | -23.5 ± 8.2 | **0.006** | 0.382 | **0.036** |
| **9** | -14.2 ± 5.7 | -16.3 ± 4.5 | 0.143 | -22.0 ± 8.8 | -23.4 ± 7.5 | 0.339 | **< 0.001** | **< 0.001** |
| **10** | -16.5 ± 3.9 | -16.2 ± 4.7 | 0.916 | -26.1 ± 7.0 | -22.5 ± 10.0 | 0.073 | **< 0.001** | **0.001** |
| **11** | -14.8 ± 6.3 | -11.7 ± 7.3 | 0.150 | -28.8 ± 10.7 | -21.1 ± 9.4 | **0.003** | **< 0.001** | **< 0.001** |
| **12** | -15.9 ± 5.4 | -13.4 ± 8.6 | 0.268 | -25.8 ± 14.1 | -28.9 ± 9.9 | 0.161 | **0.002** | **< 0.001** |
| **13** | -19.1 ± 5.4 | -19.6 ± 5.2 | 0.697 | -17.3 ± 9.7 | -27.2 ± 11.2 | **0.001** | 0.374 | **0.001** |
| **14** | -17.2 ± 4.6 | -16.0 ± 4.8 | 0.336 | -16.3 ± 5.6 | -19.0 ± 6.6 | 0.078 | 0.503 | 0.068 |
| **15** | -14.0 ± 3.6 | -15.0 ± 3.7 | 0.437 | -12.0 ± 7.3 | -19.7 ± 9.2 | **< 0.001** | **0.041** | **0.034** |
| **16** | -13.0 ± 3.7 | -13.5 ± 5.0 | 0.639 | -14.0 ± 6.5 | -17.5 ± 7.6 | 0.101 | 0.470 | **0.024** |
| **Global** | -17.0 ± 2.1 | -17.0 ± 1.7 | 0.845 | -20.2 ± 2.3 | -20.8 ± 3.2 | 0.195 | **< 0.001** | **< 0.001** |

Data are shown as mean values (in %) ± standard deviation (SD) according to the AHA-segment model [18]. Significant differences (p < 0.05) are marked bold.

**Supplemental material 4.**

**Supplemental material 4. Software Comparison – Radial_LAX_ Strain**

|  | CVI^42^ | | | TomTec | | |  |  |
| --- | --- | --- | --- | --- | --- | --- | --- | --- |
| **AHA-Segment** | **1.5 T** | **3 T** | **p value** | **1.5 T** | **3 T** | **p value** | **p value (1.5 T)** | **p value (3 T)** |
| **1** | 29.4 ± 10.8 | 24.7 ± 13.6 | 0.110 | 72.1 ± 40.3 | 72.8 ± 67.7 | 0.312 | **< 0.001** | **< 0.001** |
| **2** | 34.3 ± 14.0 | 32.0 ± 14.2 | 0.534 | 54.1 ± 70.9 | 47.7 ± 22.3 | 0.620 | **0.043** | **0.005** |
| **3** | 26.8 ± 12.3 | 26.0 ± 13.3 | 0.653 | 59.3 ± 63.5 | 76.8 ± 59.2 | 0.077 | **0.003** | **< 0.001** |
| **4** | 39.4 ± 15.9 | 46.2 ± 18.1 | 0.139 | 78.9 ± 67.5 | 69.0 ± 48.5 | 0.797 | **0.008** | **0.044** |
| **5** | 52.5 ± 26.1 | 61.1 ± 21.3 | 0.074 | 106.9 ± 69.2 | 94.7 ± 50.4 | 0.580 | **< 0.001** | **0.005** |
| **6** | 55.0 ± 23.3 | 60.8 ± 28.1 | 0.346 | 112.8 ± 86.4 | 69.6 ± 48.6 | **0.036** | **0.004** | 0.859 |
| **7** | 37.4 ± 14.5 | 37.9 ± 17.3 | 0.898 | 64.3 ± 39.8 | 42.3 ± 21.6 | **0.015** | **< 0.001** | 0.408 |
| **8** | 33.2 ± 20.6 | 40.3 ± 20.9 | 0.126 | 39.0 ± 24.2 | 48.5 ± 20.3 | **0.023** | 0.220 | 0.056 |
| **9** | 23.5 ± 12.8 | 27.0 ± 10.8 | 0.122 | 50.7 ± 23.6 | 41.8 ± 22.2 | 0.093 | **< 0.001** | **0.002** |
| **10** | 27.3 ± 10.3 | 27.6 ± 11.5 | 0.797 | 54.7 ± 43.9 | 43.4 ± 30.0 | 0.399 | **< 0.001** | **0.009** |
| **11** | 26.0 ± 13.0 | 17.3 ± 11.4 | **0.020** | 72.8 ± 37.9 | 46.0 ± 28.8 | **0.002** | **< 0.001** | **< 0.001** |
| **12** | 26.4 ± 13.6 | 23.5 ± 15.4 | 0.254 | 73.2 ± 99.7 | 40.5 ± 29.8 | **0.030** | **< 0.001** | **0.032** |
| **13** | 36.9 ± 18.8 | 38.0 ± 18.5 | 0.763 | 66.7 ± 67.4 | 64.7 ± 63.6 | 0.625 | **0.005** | 0.126 |
| **14** | 29.7 ± 11.9 | 27.0 ± 12.4 | 0.300 | 62.3 ± 52.8 | 55.5 ± 46.0 | 0.491 | **< 0.001** | **< 0.001** |
| **15** | 21.6 ± 9.4 | 23.8 ± 8.6 | 0.382 | 72.8 ± 86.1 | 64.7 ± 86.0 | 0.410 | **0.001** | 0.051 |
| **16** | 19.5 ± 7.9 | 21.2 ± 10.6 | 0.659 | 80.7 ± 92.0 | 41.5 ± 26.6 | **0.001** | **< 0.001** | **< 0.001** |
| **Global** | 29.1 ± 5.8 | 29.1 ± 4.6 | 0.792 | 77.8 ± 22.9 | 60.9 ± 14.1 | **0.002** | **< 0.001** | **< 0.001** |

Data are shown as mean values (in %) ± standard deviation (SD) according to the AHA-segment model [18]. Significant differences (p < 0.05) are marked bold.

**Supplemental material 5.**

**Supplemental material 5. Software Comparison – Circumferential Strain (using 3 short axis slices)**

|  | CVI^42^ | | | TomTec | | |  |  |
| --- | --- | --- | --- | --- | --- | --- | --- | --- |
| **AHA-Segment** | **1.5 T** | **3 T** | **p value** | **1.5 T** | **3 T** | **p value** | **p value (1.5 T)** | **p value (3 T)** |
| **1** | -24.4 ± 3.8 | -23.9 ± 4.0 | 0.678 | -24.6 ± 7.2 | -24.6 ± 5.9 | 0.854 | 0.487 | 0.554 |
| **2** | -16.3 ± 3.3 | -16.4 ± 3.1 | 0.683 | -18.5 ± 6.6 | -17.4 ± 12.8 | 0.481 | 0.222 | **0.049** |
| **3** | -11.8 ± 3.0 | -12.8 ± 4.6 | **0.033** | -20.7 ± 10.5 | -25.2 ± 7.2 | **0.048** | **< 0.001** | **< 0.001** |
| **4** | -17.4 ± 4.5 | -17.3 ± 4.0 | 0.900 | -17.3 ± 8.3 | -18.5 ± 5.1 | 0.900 | 0.503 | 0.328 |
| **5** | -22.2 ± 3.5 | -18.2 ± 3.4 | **< 0.001** | -22.9 ± 6.8 | -22.5 ± 5.8 | 0.985 | 0.866 | **0.002** |
| **6** | -23.1 ± 4.3 | -21.2 ± 3.9 | 0.077 | -26.7 ± 7.9 | -27.1 ± 6.0 | 0.753 | **0.003** | **< 0.001** |
| **7** | -19.8 ± 4.1 | -19.0 ± 4.1 | 0.597 | -18.3 ± 6.4 | -18.1 ± 7.0 | 0.669 | 0.506 | 0.410 |
| **8** | -15.9 ± 3.9 | -17.3 ± 2.7 | 0.093 | -17.1 ± 5.3 | -18.2 ± 8.3 | 0.271 | 0.374 | 0.301 |
| **9** | -14.4 ± 2.4 | -15.2 ± 2.7 | 0.211 | -18.7 ± 6.3 | -16.6 ± 7.6 | 0.521 | **0.009** | 0.073 |
| **10** | -16.5 ± 3.6 | -17.1 ± 3.0 | 0.619 | -18.2 ± 5.9 | -17.3 ± 8.3 | 0.860 | 0.180 | 0.593 |
| **11** | -20.6 ± 3.7 | -18.5 ± 3.6 | **0.022** | -22.0 ± 5.2 | -21.3 ± 6.7 | 0.826 | 0.246 | **0.018** |
| **12** | -21.4 ± 3.9 | -18.8 ± 3.8 | **0.008** | -18.7 ± 7.2 | -21.6 ± 8.2 | 0.183 | 0.064 | 0.203 |
| **13** | -22.2 ± 3.8 | -21.4 ± 3.9 | 0.337 | -22.0 ± 6.2 | -22.4 ± 6.6 | 0.637 | 0.946 | 0.123 |
| **14** | -19.4 ± 4.3 | -19.4 ± 3.8 | 0.933 | -19.8 ± 6.7 | -24.3 ± 5.5 | **0.004** | 0.982 | **0.001** |
| **15** | -23.1 ± 4.0 | -22.3 ± 4.2 | 0.531 | -26.5 ± 6.4 | -26.8 ± 5.8 | 0.900 | **0.005** | **0.001** |
| **16** | -25.4 ± 3.6 | -23.5 ± 4.8 | 0.104 | -26.7 ± 5.4 | -24.0 ± 7.3 | 0.102 | 0.352 | 0.861 |
| **Global** | -19.6 ± 2.3 | -18.8 ± 2.2 | 0.263 | -20.6 ± 2.4 | -20.9 ± 2.8 | 0.385 | 0.076 | **0.001** |

Data are shown as mean values (in %) ± standard deviation (SD) according to the AHA-segment model [18]. Significant differences (p < 0.05) are marked bold.

**Supplemental material 6.**

**Supplemental material 6. Software Comparison – Radial_SAX_ Strain (using 3 short axis slices)**

|  | CVI^42^ | | | TomTec | | |  |  |
| --- | --- | --- | --- | --- | --- | --- | --- | --- |
| **AHA-Segment** | **1.5 T** | **3 T** | **p value** | **1.5 T** | **3 T** | **p value** | **p value (1.5 T)** | **p value (3 T)** |
| **1** | 51.6 ± 17.3 | 49.4 ± 17.8 | 0.664 | 55.0 ± 31.7 | 59.0 ± 31.7 | 0.554 | 0.937 | 0.328 |
| **2** | 25.7 ± 8.3 | 26.1 ± 8.2 | 0.706 | 38.1 ± 25.3 | 41.3 ± 20.4 | 0.359 | **0.009** | **0.003** |
| **3** | 16.2 ± 5.4 | 18.8 ± 5.6 | **0.036** | 26.2 ± 17.3 | 30.7 ± 15.1 | 0.181 | **0.008** | **< 0.001** |
| **4** | 29.6 ± 12.1 | 28.4 ± 10.9 | 0.772 | 47.1 ± 27.7 | 61.0 ± 32.9 | **0.024** | **0.001** | **< 0.001** |
| **5** | 42.2 ± 12.4 | 30.1 ± 8.5 | **< 0.001** | 67.4 ± 32.7 | 82.0 ± 42.4 | 0.155 | **< 0.001** | **< 0.001** |
| **6** | 46.4 ± 15.5 | 39.1 ± 11.7 | 0.097 | 52.4 ± 33.7 | 67.3 ± 27.4 | **0.012** | 0.685 | **< 0.001** |
| **7** | 35.0 ± 12.9 | 32.7 ± 11.3 | 0.624 | 46.9 ± 23.6 | 62.9 ± 29.8 | **0.026** | **0.018** | **< 0.001** |
| **8** | 24.7 ± 9.1 | 27.6 ± 6.7 | 0.083 | 44.8 ± 19.5 | 45.6 ± 23.4 | 0.806 | **< 0.001** | **< 0.001** |
| **9** | 20.7 ± 5.1 | 22.6 ± 5.7 | 0.161 | 38.2 ± 17.0 | 49.1 ± 21.7 | **0.013** | **< 0.001** | **< 0.001** |
| **10** | 26.1 ± 8.9 | 27.2 ± 7.6 | 0.576 | 41.9 ± 21.4 | 54.5 ± 20.3 | **0.010** | **0.001** | **< 0.001** |
| **11** | 37.2 ± 10.9 | 30.8 ± 9.1 | **0.019** | 50.4 ± 20.5 | 65.2 ± 26.1 | **0.010** | **0.004** | **< 0.001** |
| **12** | 39.9 ± 12.6 | 31.9 ± 9.8 | **0.009** | 53.5 ± 22.0 | 63.0 ± 25.5 | **0.044** | **0.003** | **< 0.001** |
| **13** | 43.1 ± 13.6 | 40.4 ± 13.6 | 0.309 | 45.6 ± 19.5 | 63.1 ± 26.6 | **0.010** | 0.374 | **0.001** |
| **14** | 34.3 ± 12.2 | 33.9 ± 10.7 | 0.913 | 38.6 ± 23.6 | 66.5 ± 34.6 | **< 0.001** | 0.652 | **< 0.001** |
| **15** | 47.5 ± 15.8 | 43.6 ± 14.3 | 0.391 | 43.5 ± 23.8 | 66.0 ± 39.5 | **0.014** | 0.262 | **0.025** |
| **16** | 56.6 ± 16.6 | 48.8 ± 16.1 | 0.097 | 56.8 ± 27.3 | 67.7 ± 28.4 | 0.136 | 0.791 | **0.012** |
| **Global** | 36.0 ± 7.5 | 33.0 ± 6.2 | 0.128 | 57.4 ± 12.7 | 71.0 ± 16.4 | **0.001** | **< 0.001** | **< 0.001** |

Data are shown as mean values (in %) ± standard deviation (SD) according to the AHA-segment model [18]. Significant differences (p < 0.05) are marked bold.

**Supplemental material 7.**

**Supplemental material 7. Intra- and inter-observer reproducibility**

|  | **Intra-observer** |  |  |  | **Inter-observer** |  |
| --- | --- | --- | --- | --- | --- | --- |
| **n = 10** | **Observer 1**  **First analysis** | **Observer 1**  **Second analysis** | **p value** |  | **Observer 2** | **p value** |
| **Global longitudinal strain (%)** | -16.34 ± 1.42 | -16.40 ± 1.53 | 0.971 |  | -16.37 ± 1.36 | 0.853 |
| **Global circumferential strain (%)** | -17.20 ± 1.67 | -17.26 ± 1.52 | 0.971 |  | -16.86 ± 2.08 | 0.796 |
| **Global radial_SAX_ strain (%)** | 28.03 ± 4.48 | 28.06 ± 3.87 | 0.912 |  | 27.24 ± 5.13 | 0.853 |
| **Global radial_LAX_ strain (%)** | 27.40 ± 3.77 | 27.54 ± 4.47 | 0.853 |  | 27.36 ± 3.62 | 1.00 |

|  | **Intra-observer** |  |  |  | **Inter-observer** |  |  |
| --- | --- | --- | --- | --- | --- | --- | --- |
| **n = 10** | **ICC** | **Lower CI** | **Upper CI** |  | **ICC** | **Lower CI** | **Upper CI** |
| **Global longitudinal strain** | 0.941 | 0.759 | 0.985 |  | 0.829 | 0.273 | 0.958 |
| **Global cicrumferential strain** | 0.977 | 0.907 | 0.994 |  | 0.969 | 0.862 | 0.992 |
| **Global radial_SAX_ strain** | 0.969 | 0.874 | 0.992 |  | 0.975 | 0.889 | 0.994 |
| **Global radial_LAX_ strain** | 0.930 | 0.715 | 0.983 |  | 0.850 | 0.365 | 0.963 |

Data is shown as mean values ± standard deviations (SD)

ICC: intra-class correlation coefficient, CI: 95% confidence interval
